# A Comparative Study of Different Biological Drugs in First-Line Therapy in Crohn’s Disease: A Multicenter Descriptive Analysis

**DOI:** 10.3390/medicina62050901

**Published:** 2026-05-07

**Authors:** Andrei Gila, Carmen Monica Preda, Mircea Diculescu, Razvan Matei Bratu, Doina Istratescu, Tudor Stroie, Anca Trifan, Alina Tantau, Sandica Bucurica, Cristian George Tieranu, Horia Minea, Ana Maria Buzuleac, Lucian Negreanu, Remus Popescu, Cosmin Alexandru Ciora

**Affiliations:** 1 Gastroenterology & Hepatology Department, UMF “Carol Davila”, 020021 Bucharest, Romania; andrei_gila@yahoo.com (A.G.); mihai.diculescu@umfcd.ro (M.D.); sandica.bucurica@umfcd.ro (S.B.); cristian.tieranu@umfcd.ro (C.G.T.); lucian.negreanu@umfcd.ro (L.N.); remus-florin.popescu@drd.umfcd.ro (R.P.); cosmin.ciora@umfcd.ro (C.A.C.); 2 Gastroenterology & Hepatology Department, Fundeni Clinical Institute, 022328 Bucharest, Romania; doina.proca08@gmail.com; 3 Anatomy Department, UMF “Carol Davila”, 0200201 Bucharest, Romania; 4 Department of Gastroenterology, Faculty of Medicine, University of Medicine and Pharmacy “Grigore T. Popa”, 700115 Iasi, Romania; ancatrifan@yahoo.com (A.T.); horia.minea@yahoo.com (H.M.); buzuleac.am@gmail.com (A.M.B.); 5 Institute of Gastroenterology and Hepatology, “St. Spiridon” University Hospital, 700111 Iasi, Romania; 6 Cluj CF University Hospital, UMF “Iuliu Hatieganu” Cluj, 400347 Cluj, Romania; alitantau@gmail.com; 7 Gastroenterology & Hepatology Department, “Dr. Carol Davila” Central Military University Emergency Hospital, 010825 Bucharest, Romania; 8 Gastroenterology & Hepatology Department, Elias Emergency Hospital, 011461 Bucharest, Romania; 9 Internal Medicine Department, Emergency University Hospital, 050098 Bucharest, Romania

**Keywords:** Crohn’s disease, treatment sequencing, biologic therapy, first-line therapy, real-world data, infliximab, adalimumab, ustekinumab

## Abstract

*Background and Objectives:* Biologic agents have emerged as the most important therapeutic weapon in the treatment of Crohn’s disease (CD). The aim of this study is to describe the population of patients who received different molecules as first-line therapy and their persistence on first-line therapy. *Materials and Methods:* 622 patients from six gastroenterology departments from Bucharest, Iasi and Cluj treated between 2006 and 2024 were included in the study. A total of 87 (14%) were excluded because they received only mesalazine. A total of 535 were included in the final analysis. The main outcomes were clinical response and remission at 12 weeks of treatment and serious adverse events. *Results*: Three groups of patients were identified based on the first-line treatment: 45% of patients received adalimumab, 40% were treated with infliximab, and in 12.6% ustekinumab was given. Regarding the clinical and demographic characteristics, there are significant differences among the three patient groups in terms of age, disease extent, disease phenotype, proportion of individuals with perianal fistulas, clinical severity and the proportion of patients who underwent surgery. Median therapy duration was 48 months (6 ÷ 330). The three biologics studied (adalimumab, infliximab and ustekinumab) had a similar rate of clinical remission of 80%, the non-response rate also being similar (6.5%), but persistence on therapy at 2 years was better for adalimumab (66%) and infliximab (60%) compared to ustekinumab (45%) (*p* = 0.06). Serious adverse events leading to therapy discontinuation were more frequent in those treated with infliximab (10%) compared to adalimumab (3%) and ustekinumab (6%) (*p* = NS). *Conclusions*: There were significant baseline differences between the treatment groups, so this study represents an unadjusted comparison between the results obtained with different biologics in first-line treatment for Crohn’s disease. All three biological agents used in real life for Crohn’s disease therapy show similar efficacy, with an early clinical remission rate of approximately 80% and a non-response rate of 6.5%.

## 1. Introduction

Crohn’s disease (CD) is a chronic inflammatory disorder belonging to the group of inflammatory bowel diseases. Clinically, it follows a relapsing–remitting course, characterized by periods of remission and exacerbation. It is defined by discontinuous (“skip”) lesions that can involve any part of the gastrointestinal tract and by transmural inflammation affecting the entire bowel wall. In the absence of adequate treatment, CD may progress, leading to intestinal complications such as strictures and fistulas, as well as extraintestinal manifestations [1].

Decisions for surveillance, treatment and surgery are made using the Montreal classification, which is based on the age at onset of the disease, extension and complications (fistula/stricture) [2].

The Crohn’s Disease Activity Index (CDAI) is a standardized quantitative tool used to assess disease activity in patients with CD, both in clinical trials and in practice. It combines clinical symptoms, physical findings and laboratory values into a composite score. Clinical remission is defined as a CDAI score of less than 150 without the use of corticosteroids (i.e., corticosteroid-free remission in clinical trials) [3].

The medical management of CD is rapidly evolving, with an expanding array of therapeutic agents demonstrating efficacy in both inducing and maintaining clinical remission. Although biologic therapies are traditionally reserved for patients who fail to respond to conventional treatments such as corticosteroids and immunomodulators, recent studies suggest that early initiation of biologics—referred to as first-line biologic therapy—may be beneficial, particularly in patients with high disease activity or adverse prognostic indicators [4].

Monoclonal antibodies directed against TNF-α are potent and rapidly acting anti-inflammatory agents. Currently approved anti-TNF therapies for the treatment of CD include infliximab and adalimumab [5].

Infliximab is a monoclonal antibody targeting TNFα, which is administered intravenously [IV] at a dose of 5 mg/kg at 0, 2, and 6 weeks during induction and every 8 weeks thereafter when continued IV. This dose was established in the ACCENT I trial [6]. Regarding infliximab efficacy, two randomized controlled trials (RCTs) published 20 years ago enrolled 408 patients with clinical response at one infliximab dose [6,7]. At week 44, the probability of achieving clinical remission with infliximab [5 or 10 mg/kg every 8 weeks] over placebo was 2.15 [95% CI: 1.52–3.05]. Regarding infliximab safety, in a Cochrane network analysis the dose-adjusted OR for severe AEs for infliximab was 1.13 [95% CI: 0.79–1.62] [8].

Adalimumab is an IgG1 fully humanized anti-TNFα monoclonal antibody, recommended for the treatment of moderate-to-severe CD. The dosage of adalimumab is SC at a dose of 160 mg and then 80 mg 2 weeks after induction, then 40 mg SC every 2 weeks. A meta-analysis of three RCTs on adalimumab versus placebo, including 680 patients with moderate-to-severe CD, showed efficacy for obtaining clinical remission [RR: 3.58; 95% CI: 2.42–5.29] and clinical response [RR: 1.98; 95% CI: 1.47–2.67] at 4 weeks of therapy [9,10,11]. Safety data for the maintenance dose of adalimumab showed that the rate of adverse events was lower than that of placebo [RR: 0.57; 95% CI: 0.39–0.83], and the risk of infections was similar [RR: 0.79; 95% CI: 0.34–1.79] [9,10,11].

Ustekinumab is a fully human IgG1κ monoclonal antibody that binds to the shared p40 protein subunit of interleukin (IL) 12 and 23. According to a systematic review with meta-analysis that included four trials that enrolled 1947 patients treated with ustekinumab iv or placebo, ustekinumab showed superior clinical efficacy compared to placebo at 6 weeks in terms of both clinical response (RR: 1.56; 95% CI: 1.38–1.57) and clinical remission (RR: 1.76; 95% CI: 1.40–2.22) [12,13,14,15,16]. The pooled relative risk of adverse events on ustekinumab treatment did not differ significantly from placebo (53.8% vs. 56.1%, RR: 0.96; 95% CI: 0.89–1.03).

Vedolizumab is a monoclonal antibody that binds to integrin α_4_β_7_; blocking the α4β7 integrin results in gut-selective anti-inflammatory activity. A meta-analysis of four randomized controlled trials enrolling 1126 patients with moderate-to-severe luminal CD, treated with vedolizumab or placebo, showed the superiority of vedolizumab over placebo in terms of both clinical response (RR: 1.59, 95% CI: 1.32–1.91) and clinical remission (RR: 2.00, 95% CI: 1.51–2.66) [17,18,19,20]. The frequency of adverse events in these four trials did not differ significantly between vedolizumab and placebo (62% vs. 53.8%, RR: 1.15, 95% CI: 0.88–1.51).

This study aims to compare the efficacy and safety of various biological agents used as first-line therapy in patients with CD, using clinical outcomes as primary endpoints.

## 2. Material and Methods

A retrospective cohort study was conducted involving patients diagnosed with inflammatory bowel disease and treated in six tertiary gastroenterology departments located in Bucharest, Iași, and Cluj, Romania. The study period spanned from January 2006 to December 2024. A total of 622 patients were initially considered for inclusion. Of these, 87 patients (14%) were excluded from the final analysis as they received only 5-aminosalicylic acid (Mesalazine). Consequently, 535 patients were retained for final evaluation. All these patients were biological naïve.

All patients were retrospectively identified through review of individual medical records archived in each participating hospital. Only patients with complete clinical documentation were included. All subjects had previously signed informed consent forms permitting the use of their anonymized data for scientific purposes. The study protocol was approved by the Ethics Committee of the Fundeni Clinical Institute.

Patients who received infliximab underwent an induction regimen consisting of intravenous infusions at a dose of 5 mg/kg administered at weeks 0, 2, and 6. This was followed by a maintenance phase in which patients received 5 mg/kg every 8 weeks. In cases of secondary loss of response, the therapeutic strategy was adjusted in accordance with the national protocol for biological treatment in inflammatory bowel diseases.

Patients treated with adalimumab received induction therapy consisting of two subcutaneous doses administered at weeks 0 and 2. The induction regimen was 160 mg at week 0 followed by 80 mg at week 2. This was followed by a maintenance regimen of 40 mg subcutaneously every other week.

In the case of secondary loss of response, according to the national protocol for treatment with biological agents in inflammatory bowel diseases, therapeutic drug monitoring is performed: in the case where the serum level of adalimumab or infliximab is suboptimal, the dose is optimized; if anti-adalimumab or anti-infliximab antibodies are present, immunosuppressive treatment is added and the dose is optimized, followed by a reevaluation of the serum levels. If the serum level is optimal, a switch within the class or swap is made (the treatment is changed to a biological from another therapeutic class: ustekinumab or vedolizumab).

Patients treated with ustekinumab received induction therapy with a single intravenous (IV) infusion, weight-based as per standard protocol: 260 mg for patients ≤ 55 kg, 390 mg for those between 55 and 85 kg, and 520 mg for patients > 85 kg. This was followed by subcutaneous (SC) maintenance therapy initiated at week 8 post-induction, with a standard dose of 90 mg administered every 12 weeks. In cases of inadequate response or secondary loss of response, the dosing interval was reduced to every 8 weeks, in accordance with clinical judgment and existing guidelines.

Patients treated with vedolizumab received an induction regimen consisting of 300 mg administered intravenously at weeks 0, 2, and 6. This was followed by a maintenance phase with 300 mg IV every 8 weeks. In patients with an inadequate clinical response, the maintenance interval was shortened to every 4 weeks, based on the treating gastroenterologist’s clinical judgment.

The initiated treatments were recorded in the same category regardless of whether they were original or biosimilar drugs, because data in the literature suggest that efficacy and safety are equivalent between original and biosimilar drugs [21].

We also retrospectively recorded the use of immunosuppressants (azathioprine) concomitantly with anti-TNF agents. In Romania, according to the therapeutic protocol, treatment with anti-TNF agents, especially infliximab, is initiated concomitantly with azathioprine 1.5–2.5 mg/kg/day (which is stopped after 6–12 months), with the exception of 15–20% of patients, who present adverse reactions that require stopping azathioprine [22,23,24].

The recorded information was as follows: type of disease, age, age at diagnosis, sex, BMI, smoker, former smokers, delay between diagnosis and biological initiation, location of CD (terminal ileum and colon, colon only, or ileum/small bowel only, perianal disease), CDAI at biologic therapy initiation, surgical interventions (abdominal surgeries for CD such as intestinal and/or colonic resections and/or temporary or permanent stoma placement), calprotectin at initiation, hemoglobin (Hb) at initiation, CRP at initiation, concomitant treatment with azathioprine at the initiation of the biological agent, treatment initiation and discontinuation dates, reason for discontinuation, CDAI at 12 weeks, and serious adverse events. All patients had CDAI calculated 12 weeks after initiation of biological treatment.

### Statistical Analysis

The main outcome was clinical remission at 12 weeks of biologic treatment defined by CDAI < 150. Secondary outcomes included clinical response at 12 weeks on therapy defined by decrease in CDAI ≥ 25%, persistence of therapy at 1 year and rates of serious adverse events. Patients who failed to achieve either remission or response were classified as primary non-responders.

Statistical analysis was performed using SPSS version 20.0 (IBM Corp., Armonk, New York, NY, USA). Data normality was evaluated using the Kolmogorov–Smirnov test. Quantitative variables with a normal distribution were presented as mean ± standard deviation, while non-normally distributed variables were reported as median with minimum and maximum values. For group comparisons, the independent sample *t*-test was applied to normally distributed data, and the Mann–Whitney U test to non-normally distributed data. Categorical variables were reported as percentages and analyzed using the Pearson chi-square test when expected cell counts were adequate, while Fisher’s exact test was applied when expected frequencies were small. All tests were two-sided, with a *p*-value of less than 0.05 considered statistically significant.

## 3. Results

### 3.1. Description of Patients Included in the Study

Of the 535 patients included in the study, 55% remained on first-line treatment, 33% required a switch to a second-line biologic, 9% had three lines of biologic treatment, 3% were exposed to four lines of treatment and approximately 1% required even five lines.

These patients were followed for a median period of 42 months (minimum 6, maximum 330 months).

**Figure 1:**
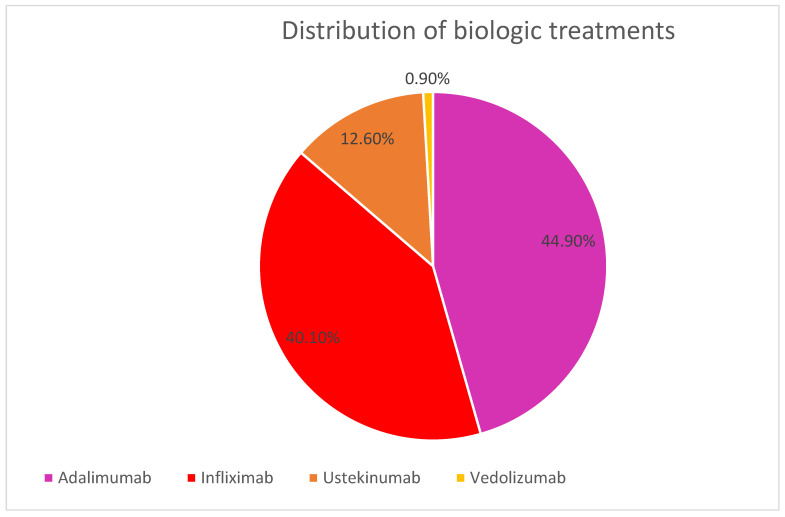
Distribution of our CD patients according to first-line biologic therapy.

In the first line of treatment these patients received the following molecules: 242 (44.9%) adalimumab, 216 (40.1%) infliximab, 68 (12.6%) ustekinumab, and only five (0.9%) vedolizumab, as represented in Figure 1. We divided the patients into four groups depending on the first-line biological treatment followed, and their demographic and clinical features are showed in Table 1.

According to the data presented in Table 1, these subjects had a mean age of 33 years (range 16–69 years), were predominantly A2 (67%), L3 (42%), and B1 (53%), and 40% were smokers, with an average CDAI of 245 points at the start of treatment. Biological treatment was initiated on average 6 months after diagnosis.

The study showed statistically significant differences between the biological treatment groups (infliximab, adalimumab, ustekinumab, and vedolizumab), highlighting the distinct clinical profiles of patients receiving these treatments. The age of patients varied considerably from one group to another (*p* < 0.001). Patients treated with ustekinumab had a higher median age (43 years), while those in the infliximab group were younger (median age 32 years). The gender distribution did not show any statistically significant differences (*p* = 0.107), with the proportion of men varying between 47.1% and 62%.

The proportion of active smokers differed significantly between groups (*p* = 0.008), with the highest percentage observed in patients treated with infliximab (44.4%) and adalimumab (39.7%), compared with only 20% in the vedolizumab group. Body mass index (BMI) did not differ significantly between groups (*p* = 0.104), with median values ranging from 21.45 to 23.91.

The distribution of disease extent (L1/L2/L3) showed significant differences (*p* = 0.001). All patients treated with vedolizumab had extensive disease (L3), while in the adalimumab group, the distribution was more balanced between L1, L2, and L3. The disease phenotype (B1/B2/B3) was significantly associated with the type of treatment: B1 (uncomplicated inflammation) was more common in the ustekinumab-treated group (79.4%)—*p* < 0.001, while B2 (stricturing) and B3 (penetrating) were more common in patients treated with adalimumab and infliximab. The presence of perianal disease was significantly higher in the infliximab-treated group (37%) than in the adalimumab-treated group (13.2%) (*p* < 0.001).

As seen in Table 2, disease activity at baseline, as expressed by the CDAI score, differed between groups (*p* = 0.021). Vedolizumab was administered to patients in clinical remission (median CDAI = 36), in contrast to the other groups, where medians were greater than 230. CRP rates at biologic therapy initiation varied considerably between groups (*p* < 0.001), with the highest values observed in patients in the vedolizumab and infliximab groups. Individuals treated first-line with adalimumab and infliximab needed prior abdominal surgery more frequently (36.4%/33.3%) than those who received first-line ustekinumab (15.2%), once again emphasizing the individualization of treatment by prescribers, depending on the severity of the disease. These results highlight the heterogeneity of clinical characteristics depending on the therapeutic strategy chosen in first-line biological treatment.

### 3.2. Retrospective Comparative Study of the Efficacy and Safety of Different First-Line Biological Treatments in Patients with CD Included in the Study

**Figure 2:**
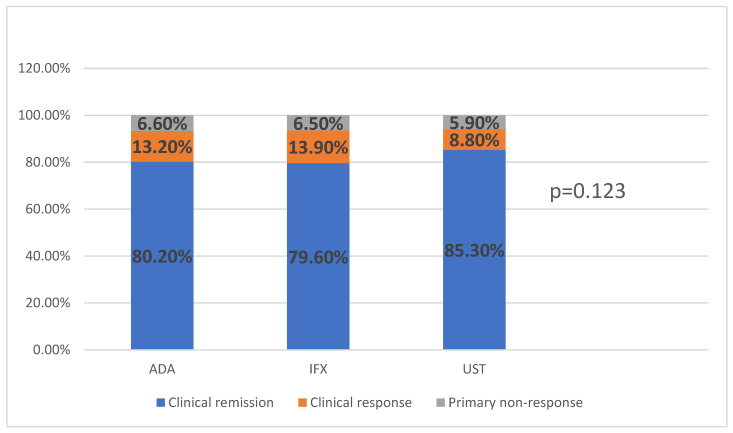
Response to therapy after 12 weeks of treatment, according to the type of biologic therapy.

A comparative analysis of the efficacy of the three biological agents—adalimumab, infliximab, and ustekinumab—revealed similar rates of clinical remission among patients included in the study, as seen in Figure 2. Patients treated in first line with vedolizumab were excluded from this analysis because their number was very small (5).

The proportion of patients who achieved clinical remission was similar across the three patient groups (adalimumab, infliximab and ustekinumab), as shown in Figure 2. However, these findings represent unadjusted comparisons and should be interpreted with caution given the significant baseline differences between groups, including age, disease behavior, and presence of perianal disease. Therefore, these results should be considered descriptive rather than indicative of comparable efficacy.

**Figure 3:**
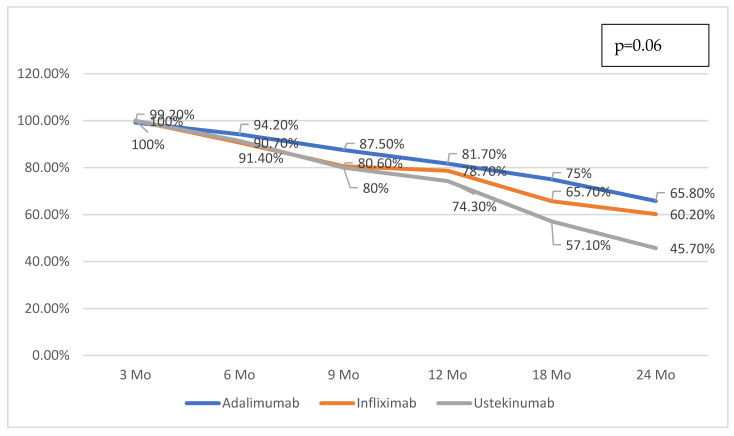
Persistence on different biologic therapy on first line in patients with CD included in the study.

A key aspect of evaluating biological treatment in chronic inflammatory bowel diseases is long-term treatment maintenance. We evaluated treatment persistence comparing the three molecules over 2 years of biological treatment, and the persistence rates are presented in Figure 3. However, these findings should be interpreted with caution, as the duration of follow-up differed substantially between groups. Patients treated with ustekinumab had a shorter median follow-up (18 months), resulting in a higher degree of right censoring at the 24-month time point. Therefore, no firm conclusions regarding comparative drug persistence can be drawn from these data.

During follow-up, 54% of those initiated on infliximab, 53% of those initiated on adalimumab, and 19% of those initiated on ustekinumab discontinued their first-line biological therapy. Of course, these differences are primarily due to the duration of follow-up: those who received first-line anti-TNF monoclonal antibody treatment had a median follow-up of 48 months, while those treated with ustekinumab had a median follow-up of 18 months.

**Figure 4:**
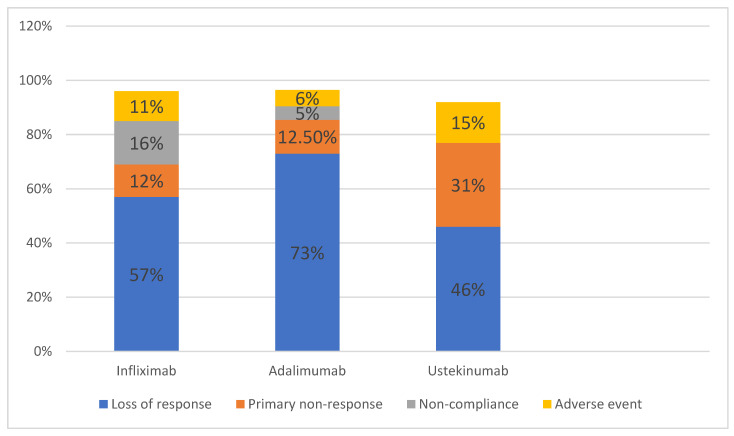
Reasons for treatment discontinuation among the 3 patient groups studied.

Figure 4 illustrates the reasons for discontinuation of biologic therapy across the three treatment groups analyzed. As shown, the most common cause for stopping biologic treatment in patients with CD was secondary loss of response, followed by primary non-response and adverse events. Notably, non-compliance was relatively uncommon, occurring in 5% of patients receiving first-line adalimumab and 11% of those treated with infliximab. The majority of adverse events reported among people treated with infliximab in this cohort were bacterial infections (7 of 13 adverse events reported), mainly tuberculosis. One case of colon cancer was also reported. In five cases, infliximab treatment had to be discontinued due to severe allergic reactions.

Seven patients treated with adalimumab discontinued treatment due to adverse events: three cases of severe bacterial infection, two cases of severe allergic reactions, one case of paradoxical psoriasis and one case of gastric cancer (unrelated to biological treatment).

Of the 13 patients treated with ustekinumab who discontinued treatment, only two experienced adverse events, both paradoxical psoriasis.

### 3.3. Predictive Factors for Clinical Remission at 12 Weeks According to Different Biological Agents Studied

In the infliximab-treated cohort, several baseline characteristics were associated with clinical remission, as seen in Table 3. Younger age is a negative predictive factor, while smoking cessation and a higher body mass index are positive predictive factors for clinical remission in patients treated with infliximab. Other negative predictive factors for the lack of clinical remission are the presence of stricturing disease, perianal fistulas, an increased time interval from the time of diagnosis to the initiation of infliximab, as well as very high clinical severity of the disease and very high fecal calprotectin levels. In contrast, the presence of ileal involvement is associated with a better clinical response to infliximab.

In the adalimumab-treated cohort, quitting smoking and disease behavior were significantly associated with clinical remission, as seen in Table 4. The lack of stricturing phenotype of the disease, the presence of the inflammatory form of the disease and lower CRP levels are also positive predictive factors for clinical remission.

We also evaluated predictors of clinical remission in patients treated with ustekinumab, as presented in Table 5. Notably, the number of patients treated with ustekinumab was considerably smaller than those treated with infliximab or adalimumab. The only positive predictor identified was male sex, which was associated with clinical remission. The analyses of predictive factors for treatment response were based on univariate comparisons and should be considered exploratory. Given the potential for confounding and the relatively small sample size in some subgroups, particularly in the ustekinumab cohort, these findings may be subject to type II error and should be interpreted cautiously.

## 4. Discussions

This multicenter retrospective analysis highlights the real-world efficacy and tolerability of three widely used biologics, infliximab, adalimumab, and ustekinumab, when used as first-line treatments in CD. Of the total of 535 patients included in our cohort, only five received first-line vedolizumab, and therefore these individuals were not included in the statistical analysis. Most of them received adalimumab (242 patients, 44.9%), 216 (40.1%) were treated with infliximab, and only 68 (12.6%) received first-line biological treatment with ustekinumab. This difference derives primarily from the fact that Romanian patients have had access to infliximab since 2005 and to adalimumab since 2007, while ustekinumab could only be prescribed in Romania from 2021 [25]. That is why an important source of bias in our study is related to the temporal availability of biologic therapies. Over this period, the management of CD has evolved considerably, including the adoption of treat-to-target strategies, improved monitoring, and earlier treatment optimization. These differences may have influenced both treatment selection and outcomes, introducing a potential era effect that could not be fully accounted for in this retrospective analysis.

Our results contribute to the growing body of comparative efficacy research, while providing information specific to a cohort in Central and Eastern Europe. It is important to verify the data coming from clinical trials and network meta-analysis through real-life studies, including retrospective studies of efficacy and safety.

The study showed that the three agents achieved a similar early complete clinical response rate of approximately 80% and a non-response rate of 6.5%, which is consistent with previous European and North American data suggesting comparable short-term efficacy [22,23,24,26,27,28,29,30,31,32]. However, in our study, the number of patients treated with ustekinumab (68) was much smaller than the number of those treated with infliximab and adalimumab (216/242), which represents a limitation of the study. Although similar crude rates of early clinical response were observed across the three biologic agents, these findings must be interpreted with caution. The treatment groups were characterized by substantial baseline differences, reflecting real-world treatment allocation. Patients receiving infliximab were generally younger and had more severe disease phenotypes, including a higher prevalence of penetrating and perianal disease, and required previous abdominal surgery much more frequently, whereas those treated with ustekinumab had milder inflammatory disease and were older. These imbalances introduce significant confounding by indication, limiting the ability to draw direct comparative conclusions regarding efficacy. Our retrospective study is a descriptive study, aiming to describe the prescription pattern and personalization of treatment by doctors working in Romania in the field of IBD and the results obtained with the three molecules, which are still the most prescribed in Romania in the treatment of Crohn’s disease, even though the therapeutic arsenal has diversified.

It should be noted that in all these retrospective studies there are statistically significant differences between the demographic and disease characteristics of the patients; as a general rule, patients with a more severe clinical and evolutive phenotype (younger cases, fistulizing forms, patients with perianal fistulas) receive infliximab as the first line of treatment [22,23,24,26,27,28,29,30,31,32]. This is also the situation with the patients included in our study, reflecting the personalization of biological treatment in CD. However, this should not prevent us from studying the comparative efficacy and safety of different molecules for these patients.

Most data in the literature suggest a persistence on first-line biologics in CD of 40–45%, but the patients in our study group had a better persistence on treatment: 55% in this cohort with a median follow-up of 42 months [22,23,24,26,27,28,29,30,31,32].

Regarding treatment persistence according to first-line biologic therapy, our descriptive study suggests a greater persistence with first-line anti-TNF agents compared to ustekinumab, but this is an unadjusted analysis in substantially non-equivalent groups, so these results should be interpreted with caution. At 2 years, 66% of patients treated with adalimumab remained on therapy, compared to 60% of those receiving infliximab and 46% of those treated with first-line ustekinumab (*p* = 0.06). The observed differences in treatment persistence between biologic agents should also be interpreted cautiously. The shorter follow-up duration in the ustekinumab group results in a higher proportion of censored observations at later time points, particularly at 24 months. As a result, direct comparisons of drug survival between groups may be biased, and these findings should be considered descriptive rather than conclusive.

The study published by Gil-Candel M et al. shows similar drug survival between infliximab and adalimumab, and the main cause of treatment discontinuation is secondary loss of response, results similar to those obtained in our retrospective study [33]. Therapeutic drug monitoring was associated with higher drug survival for both first- and second-line anti-tumor necrosis factor treatments in the study by Gil-Candel M et al. [33].

In a South Korean nationwide database large study published by Koo HM et al., which included 13,087 patients with inflammatory bowel diseases, ustekinumab had significantly better persistence than infliximab and adalimumab, suggesting that there may be very large differences between the studied populations [34].

Infliximab was associated with a higher rate of serious adverse events (10%) compared with adalimumab (3%) and ustekinumab (6%), although these differences are not statistically significant. The similar study published by Mateescu RB et al. also found a similar profile of severe adverse events in patients with inflammatory bowel disease, regardless of the ongoing biological treatment [24]. Tursi A et al. found a significantly higher rate of adverse events in patients treated with infliximab compared to those receiving adalimumab [21].

However, it is essential to interpret these safety signals with caution, as patients treated with infliximab also had a more severe form of the disease, which could skew the risk profile.

In the literature, the predictive factors for a good clinical response to infliximab are infliximab trough levels (TLs), absence of anti-infliximab antibodies, concomitant immunosuppressants and endoscopic healing. In our cohort study, the majority of patients who were treated with infliximab received concomitant azathioprine (except those who did not tolerate azathioprine). Therapeutic drug monitoring was performed for patients on anti-TNF therapy according to the national protocol for treatment with biological agents in inflammatory bowel diseases. In the infliximab-treated group, older age, quitting smoking, absence of perianal disease, a less severe active clinical phenotype, lower fecal calprotectin levels and ileal (L1) disease location were significantly associated with early clinical remission. These factors were also identified by Arnott et al. in their study [35], while Vermeire S et al. found that, on the contrary, young age and colonic extension of the disease correlate with a better clinical response to infliximab [36]. Our data showed that for adalimumab, not smoking and an inflammatory (B1) disease behavior predicted better outcomes. Other studies in the literature have identified the following predictive factors for lack of response to adalimumab: presence of extraintestinal manifestations, low drug concentration, longer disease duration, presence of strictures and low level of CRP at therapy initiation [37,38,39,40].

We looked for predictors of response to ustekinumab in univariate analysis, although the number of patients treated with ustekinumab in our study was relatively small (68). The only predictor we identified for early clinical remission was male gender. This finding should be interpreted with caution, as the analysis was based on univariate comparisons and the number of non-responders in the ustekinumab group was very small. Therefore, this association may reflect a sample size artifact rather than a true independent predictor. Other studies report female gender, lower clinical severity of disease, ileocolonic disease, no prior surgery and uncomplicated phenotype [41,42,43,44].

This study has several notable strengths. It is based on a large real-world cohort of 535 patients with CD who had never received biological treatment, giving it high statistical power and clinical relevance. Its multicenter design, involving six tertiary gastroenterology centers in Romania, strengthens the generalizability of the results to similar healthcare settings. Importantly, the study focuses on first-line biologic treatment, a relatively unexplored area, thus providing valuable insights into early treatment strategies for CD. By directly comparing three widely used biologic agents, infliximab, adalimumab, and ustekinumab, the analysis provides practical data for therapeutic decision-making. The inclusion of long-term follow-up, with an assessment of treatment persistence at two years, allows for the evaluation of long-term efficacy and tolerance. In addition, the study records a wide range of baseline demographic, clinical, and biological parameters, allowing for the exploration of predictors of treatment response.

Despite its strengths, this study also has some limitations. An important limitation of this study is the lack of adjustment for baseline differences between treatment groups. Due to the retrospective design and uneven distribution of patients across treatment arms, advanced statistical methods such as propensity score matching or multivariable modeling were not applied, which limits the ability to draw causal inferences. Consequently, the results should be interpreted as hypothesis-generating rather than definitive comparisons of treatment efficacy. Additionally, the relatively small number of patients in certain subgroups, particularly those treated with ustekinumab and vedolizumab, may limit statistical power and the precision of estimates. This is especially relevant for the analysis of predictive factors, where small sample sizes increase the likelihood of type II error. Since all data were collected from Romanian institutions, their generalizability to other populations or healthcare systems may be limited. It would have been extremely informative to be able to analyze in this cohort the evolution of some objective parameters during treatment, namely CRP, fecal calprotectin, and endoscopic severity, but, unfortunately, these parameters were not consistently recorded during the follow-up of the patients, and therefore they were excluded from the statistical analysis.

## 5. Conclusions

Overall, this concrete analysis confirms that, although anti-TNF treatments remain highly effective, ustekinumab may offer comparable short-term results and greater safety in certain patients. Prospective randomized studies are needed to confirm these results and refine the algorithms for biological treatment of CD.

## Figures and Tables

**Table 1:** The most important demographic and clinical characteristics of patients with CD in the study group, depending on the first-line biological treatment.

GROUP		All Patients	Vedolizumab	Ustekinumab	Infliximab	Adalimumab	*p*-Value
		No. (%)	No. (%)	No. (%)	No. (%)	No. (%)	
No		535 (100%)	5 (0.9%)	68 (12.6%)	216 (40.1%)	242 (44.9%)	
Age mean		33	36	43	32	33	<0.001
Sex	M	315 (58.9%)	3 (60%)	32 (47.1%)	134 (62%)	142 (58.7%)	0.107
	F	220 (41.1%)	2 (40%)	36 (52.9%)	82 (38%)	100 (41.3%)	
Smokers		215 (40.2%)	1 (20%)	18 (26.5%)	96 (44.4%)	96 (39.7%)	0.008
Former smokers		106 (19.7%)	0 (0%)	8 (11.8%)	44 (20.4%)	54 (22.3%)	0.221
BMI mean (kg/m^2^)		23.38	21.45	23.91	23.45	23.05	0.104
Age at diagnosis (A)	A1	14 (3%)	0 (0%)	0 (0%)	10 (5.1%)	4 (1.9%)	0.002
	A2	305 (65.9%)	5 (100%)	26 (50%)	136 (68.7%)	138 (66.3%)	
	A3	144 (31.1%)	0 (0%)	26 (50%)	52 (26.3%)	66 (31.7%)	
Disease location (L)	L1	154 (28.6%)	0 (0%)	30 (44.1%)	34 (15.7%)	88 (36.4%)	0.001
	L2	154 (28.6%)	0 (0%)	14 (20.6%)	86 (39.8%)	54 (22.3%)	
	L3	227 (42.1%)	5 (100%)	24 (35.3%)	96 (44.4%)	100 (41.3%)	
Disease behavior (B)	B1	283 (52.9%)	3 (60%)	54 (79.4%)	102 (47.2%)	122 (50.4%)	<0.001
	B2	164 (30.4%)	0 (0%)	12 (17.6%)	54 (25%)	96 (39.7%)	<0.001
	B3	88 (16.4%)	2 (40%)	0 (0%)	64 (29.6%)	22 (9.1%)	<0.001
Perianal disease		114 (21.3%)	2 (40%)	0 (0%)	80 (37%)	32 (13.2%)	<0.001
Time from diagnosis to biologic initiation (mean) (months)		6	1	5.5	6	6	0.001
Surgery before treatment initiation		170 (31.9%)	0	10 (15.2%)	72 (33.3%)	88 (36.4%)	0.004
Colonic/intestinal resections before treatment initiation		162 (30.3%)	0	10 (15.2%)	68 (31.5%)	84 (34.7%)	0.005
Permanent/temporary stoma		11 (2.1%)	0	1 (1.5%)	4 (1.9%)	6 (2.5%)	0.01
The average no of surgical interventions in operated patients		1.1	0	1.2	1.1	1.1	0.42
Concomitant immunomodulator (azathioprine) at initiation of 1st-line biologic		231 (43.2%)	0 (0%)	0 (0%)	156 (72.2%)	75 (31.0%)	<0.001

**Table 2:** Clinical severity of the disease according to the CDAI score, C-reactive protein (CRP) and fecal calprotectin levels compared in the 4 patient groups.

GROUP	All Patients	Vedolizumab	Ustekinumab	Infliximab	Adalimumab	*p*-Value
INITIAL CDAI Mean	245	36	278.5	230	245	0.021
INITIAL CRP Mean (mg/dL)	11.2	25	8	21	9.3	<0.001
INITIAL Calprotectin Mean (mcg/g)	650	234	506	467	667	0.654

**Table 3:** Predictive factors for clinical remission in patients treated with infliximab.

		Clinical Remission = YES (152)	Clinical Remission = NO (44)	*p*-Value
**AGE Mean**		**32**	**25.5**	***0.014***
SEX No (%)	M	106 (69.7%)	28 (63.6%)	*0.863*
Smokers No (%)		74 (43%)	22 (50%)	*0.497*
**Former Smokers** No (%)		**40 (23.3%)**	**4 (9.1%)**	***0.038***
**BMI Mean** (kg/m^2^)		**23.45**	**22.4**	***0.007***
**L Subgroups** No (%)	**L1**	**32 (18.6%)**	**2 (4.5%)**	*0.029*
	L2	64 (37.2%)	22 (50%)	
	L3	76 (44.2%)	20 (45.5%)	
**Disease Behavior** No (%)	B1	84 (48.8%)	18 (40.9%)	*0.399*
	**B2**	**36 (20.9%)**	**18 (40.9%)**	***0.01***
	B3	54 (31.4%)	10 (22.7%)	*0.355*
**Perianal Disease** No (%)		**54 (31.4%)**	**26 (59.1%)**	***0.001***
**Time Diagnosis to Biologic Mean (Months)**		**7**	**36**	***<0.001***
**INITIAL CDAI Mean**		**231**	**343.5**	***<0.001***
INITIAL CRP Mean (mg/dL)		21	12	*0.273*
**INITIAL Calprotectin Mean** (mcg/g)		**598**	**1000**	***0.054***
INITIAL Hemoglobin Mean (g/dL)		12.6	11.5	*0.07*
Extraintestinal Manifestations No (%)		24 (14.1%)	7 (15.9%)	*0.811*

**Table 4:** Predictive factors for complete clinical remission in patients treated with adalimumab.

		Clinical Remission = YES (194)	Clinical Remission = NO (48)	*p*-Value
AGE Mean		35	34	0.733
SEX No (%)	M	118 (60.8%)	24 (50%)	0.192
**Smokers** No (%)		**70 (36.1%)**	**26 (54.2%)**	**0.031**
Former Smokers No (%)		44 (22.7%)	10 (20.8%)	0.784
BMI Mean (kg/m^2^)		23.43	22.86	0.181
L Subgroups No (%)	L1	76 (39.2%)	12 (25%)	0.169
	L2	42 (21.6%)	12 (25%)	
	L3	76 (39.2%)	24 (50%)	
Disease Behavior No (%)	**B1**	**106 (54.6%)**	**16 (33.3%)**	**0.01**
	**B2**	**66 (34%)**	**30 (62.5%)**	**<0.001**
	B3	20 (10.3%)	2 (4.2%)	0.264
Time Diagnosis to Biologic Mean (Months)		11	18.5	0.558
INITIAL CDAI Mean		250	220	0.453
**INITIAL CRP Mean** (mg/dL)		**7**	**19.9**	**0.01**
INITIAL Calprotectin Mean (mcg/g)		547	784	0.432
INITIAL Hemoglobin Mean (g/dL)		12.8	12.35	0.723
Extraintestinal Manifestations No (%)		33 (17.1%)	8 (17%)	1

**Table 5:** Predictive factors for clinical remission in patients treated with ustekinumab.

		Clinical Remission = Y (58)	Clinical Remission = N (10)	*p*-Value
AGE Mean		39.5	39.5	1
**SEX** No (%)	**M**	**32 (55.2%)**	**0 (0%)**	**<0.001**
Smokers No (%)		18 (31%)	0 (0%)	0.06
Former Smokers No (%)		8 (13.8%)	0 (0%)	0.215
BMI Mean (kg/m^2^)		23.8	25.71	0.299
L Subgroups No (%)	L1	26 (44.8%)	4 (40%)	0.737
	L2	12 (20.7%)	2 (20%)	
	L3	20 (34.5%)	4 (40%)	
Disease Behavior No (%)	B1	44 (75.9%)	10 (100%)	0.108
	B2	12 (20.7%)	0 (0%)	0.189
	B3	2 (3.4%)	0 (0%)	0.554
Time Diagnosis to Biologic Mean **(months)**		5	5	0.754
INITIAL CDAI Mean		260	189	0.066
INITIAL CRP Mean (mg/dL)		7	5.9	0.467
INITIAL Calprotectin Mean (mcg/g)		500	655	0.16
INITIAL Hemoglobin Mean (g/dL)		13	12.2	0.723
Extraintestinal Manifestations No (%)		14 (24.1%)	2 (20%)	1.000

## Data Availability

Data is unavailable due to privacy restrictions.
